# Recurring septic shock in a patient with blunt abdominal and pelvic trauma: how mandatory is source control surgery?: a case report

**DOI:** 10.1186/s13256-017-1206-6

**Published:** 2017-02-22

**Authors:** Antonella Frattari, Giustino Parruti, Rocco Erasmo, Luigi Guerra, Ennio Polilli, Rosamaria Zocaro, Giuliano Iervese, Paolo Fazii, Tullio Spina

**Affiliations:** 1 Unit of Anaesthesia and Intensive Care, Santo Spirito Hospital, Via Fonte Romana 8, Pescara, Italy; 2 Unit of Infectious Diseases, Santo Spirito Hospital, Via Fonte Romana 8, Pescara, Italy; 3 Unit of Orthopedics and Traumatology, Santo Spirito Hospital, Via Fonte Romana 8, Pescara, Italy; 4 Unit of Microbiology, Santo Spirito Hospital, Via Fonte Romana 8, Pescara, Italy

**Keywords:** Septic shock, Intensive assistance, Control surgery, Case report

## Abstract

**Background:**

In critically ill patients with colonization/infection of multidrug-resistant organisms, source control surgery is one of the major determinants of clinical success. In more complex cases, the use of different tools for sepsis management may allow survival until complete source control.

**Case presentation:**

A 42-year-old white man presented with traumatic hemorrhagic shock. Unstable pelvic fractures led to emergency stabilization surgery. Fever ensued with diarrhea, followed by septic shock. Two weeks later, an abdominal computed tomography scan revealed suprapubic and ischiatic abscesses at surgical sites, as well as dilated bowel. Debridement of both surgical sites, performed with vacuum-assisted closure therapy, yielded isolates of carbapenem and colistin-resistant *Klebsiella pneumoniae*. Antibiotic treatment was de-escalated after 21 days; 4 days later fever, leukocytosis, hypotension and acute renal failure relapsed. Blood purification techniques were started, for the removal of endotoxin and inflammatory mediators, with sequential hemodialysis. Clinical improvement ensued; blood cultures yielded *Candida albicans* and multidrug-resistant *Acinetobacter baumannii*; panresistant carbapenemase-producing *Klebsiella pneumoniae* grew from wound swabs. In spite of shock reversal, our patient remained febrile, with diarrhea. Control blood cultures yielded *Candida albicans*, *Acinetobacter baumannii* and carbapenem-resistant *Klebsiella pneumoniae*. His abdominal pain increased, paralleled by a right flank palpable mass. Colonoscopy revealed patchy serpiginous ulcers. At exploratory laparotomy, an inflammatory post-traumatic pseudotumor of his right colon was removed. Blood cultures turned negative after surgery. Septic shock, however, relapsed 4 days later. A blood purification cycle was repeated and combination antimicrobial therapy continued. Surgical wounds and blood cultures were persistently positive for carbapenem-resistant *Klebsiella pneumoniae*. Removal of pelvic synthesis media was therefore anticipated. Three weeks later, clinical, microbiological, and biochemical evidence of infection resolved.

**Conclusions:**

High quality intensive assistance for sepsis episodes needs a clear plan of cure, aimed to complete infection source control, in a complex multidisciplinary interplay of specialists and intensive care physicians.

## Background

Sepsis is a well-recognized factor contributing to poor outcome after severe traumatic injury [1]. Independent risk factors for post-traumatic sepsis are massive transfusion of packed red blood cells, high Injury Severity Score (ISS), surgery, and prolonged intensive care unit (ICU)/hospital stay [2]. Sepsis is often complicated in its course in such patients, mainly because of the persistence of predisposing factors and multidrug-resistant (MDR) bacteria involvement, in particular carbapenem-resistant *Klebsiella pneumoniae* (CRKP) and other difficult-to-treat Gram-negative microorganisms [3–5]. To manage sepsis and septic shock in this setting, complex strategies and interventional bundles have been deployed in recent years [6]. These include combination antimicrobial therapy, infection source control, and other intensive supportive therapies, whose role is well established; blood purification techniques may play an additional role [6–11]. In this scenario, it is useful to describe even single complicated cases, such as the one reported here, to outline and pinpoint the role of each of these tools in the global management of patients with sepsis.

## Case presentation

A 42-year-old white man without pre-existing comorbidities was transferred to our unit on 8 September 2014. He was involved in a car crash on 30 August 2014 with traumatic hemorrhagic shock and an ISS of 28. Shock resuscitation according to Advanced Trauma Life Support (ATLS) guidelines and massive transfusion policy were immediately started [12]. Upon hemodynamic stabilization, a total body computed tomography (CT) scan confirmed unstable pelvic fractures and right flank mesenteric bleeding, without clear evidence of intestinal perforation. Damage control surgery of his pelvic fractures was performed by positioning external fixators [13, 14]. Transfer to our unit was then planned for definitive osteosynthesis.

On arrival he had fever and profuse diarrhea, leukocytosis, normal procalcitonin (PCT), elevated C-reactive protein (CRP), lactate 0.9 mmol/L, an Acute Physiology and Chronic Health Evaluation (APACHE) II score of 11, and a Sequential Organ Failure Assessment (SOFA) score of 3; his Predisposition, Infection, Response, and Organ Dysfunction (PIRO) score was 5 [15]. He wore a tracheostomy and was mechanically ventilated on analgosedation; he was left on parenteral nutrition and a minimal enteral feeding was started.

**Fig. 1:**
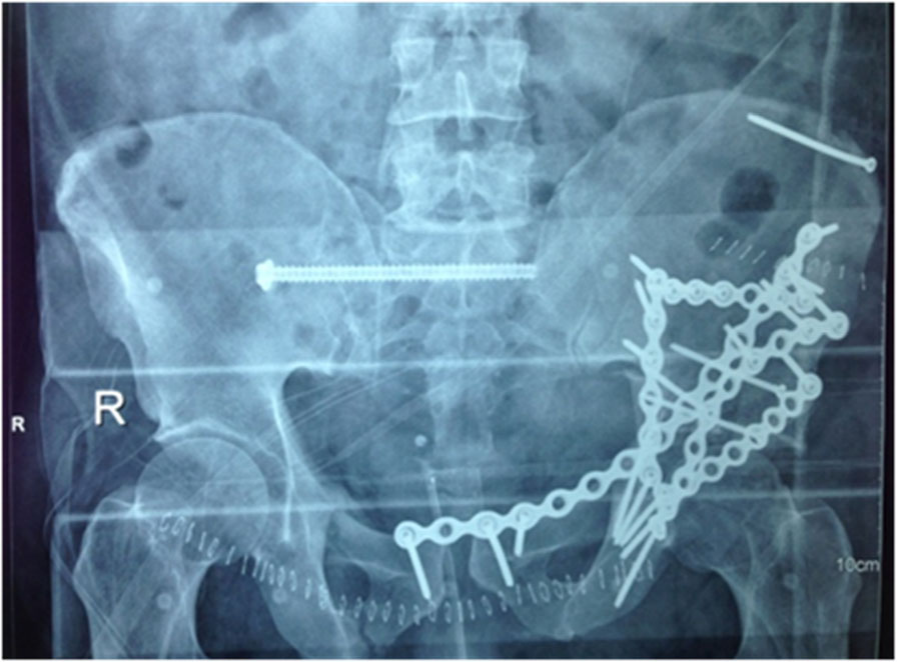
Pelvic fracture of the patient after the intervention of osteosynthesis

Chest X-rays detected a right basal infiltrate. Orthopedic surgery was postponed. After thorough microbiological sampling, immune chromatography for *Clostridium difficile* was negative, rectal swabs grew MDR *Acinetobacter baumannii*, whereas blood cultures yielded coagulase-negative staphylococci. His antibiotic therapy was modified as described here and in Table 1. As external fixators poured purulent secretions, on day 15 he underwent definitive pelvic osteosynthesis in spite of persistent fever and diarrhea (Fig. 1). Septic shock ensued 24 hours after surgery, with oliguria, leukocytosis, PCT 4.39 ng/ml, CRP 46.6 mg/L, and lactate 1.17 mmol/L; his SOFA score rose to 6. After adequate fluid resuscitation, norepinephrine was added for persistent hypotension, based on data of hemodynamic monitoring (cardiac output and stroke volume variation using PiCCO Plus monitoring system) and trends of central venous saturation of oxygen (ScVO_2_). Low doses of steroids were prescribed for the first 3 days. Microbiological sampling included blood cultures, quantitative culture of tracheal secretions, and culture of urine [6]. Blood cultures were negative; urine and tracheal aspirate samples were positive for CRKP and MDR *A. baumannii*.

**Table 1:** Overview of antimicrobial association treatments used in this patient

Days of hospitalization in intensive care	Combination therapy	Doses	Microbiological data	Site of infection
1–7	Piperacillin/tazobactam Levofloxacin Linezolid	LD 4.5 gr + 4.5 gr/6 hour 500 mg/12 hour 600 mg/12 hour	Empirical regimen Negative microbiological data from the dispatching ward	Lung Intestine
7–21	Daptomycin Tigecycline Meropenem Anidulafungin	8 mg/Kg (1 gr/day) LD 200 mg + 100 mg/12 hour LD 2 gr + 2 gr/8 hour LD 200 mg + 100 mg/day	Blood cultures: *Staphylococcus hominis* and *Staphylococcus epidermidis * Rectal swab: MDR *Acinetobacter baumannii*	Pelvic insertion sites Intestine Bloodstream
21–32	Colimycin (colistin) Tigecycline Meropenem Daptomycin	LD 9 MU + 4.5 MU/12 hour intravenous 1 MU/8 hour nebulized 100 mg/12 hour 2 gr/8 hour 1 gr/day	Blood cultures: negative Urine and tracheal aspirate: *KPC-producing Klebsiella pneumoniae* and MDR *A. baumannii * Wounds swab: *KPC-producing K. pneumoniae* panres	Lung Urinary tract Surgical site
32–36	Meropenem Tigecycline	2 gr/8 hour 100 mg/12 hour	Blood cultures: *S. Epidermidis* (1 broth) Tracheal aspirate: *KPC-producing K. pneumoniae* panres	Intestine Surgical site
36–72 36–68 36–48 48–68	Colimycin (colistin) Tigecycline Meropenem Rifampicin Anidulafungin Amphotericin	LD 9 MU + 4.5 MU/12 hour intravenous + 1 MU/8 hour nebulized 100 mg/12 hour 2 gr/8 hour 900 mg/day LD 200 mg +100 mg/day 5 mg/kg each second day	SeptiFast: *A. baumannii* and *Candida albicans * Tracheal aspirate: *A. baumannii* and *KPC-producing K. pneumoniae * Blood cultures: *C. albicans* and *A. baumannii* Wound swabs: *KPC-producing K. pneumoniae* panres Control blood cultures (3 lots): *C. albicans A. baumannii* and *KPC-producing K. pneumoniae* panres	Bloodstream Surgical site Intestine
72–77	Meropenem Tigecycline	2 gr/8 hour 100 mg/12 hour	Wound swabs: *KPC-producing K. pneumoniae* panres	Surgical site
77–132	Colimycin (colistin) Meropenem Ertapenem Tigecycline	LD 9 MU + 4.5 MU/12 hour 2 gr/6 hour 1 gr/12 hour 100 mg/12 hour	Surgical wounds and blood cultures: *KPC-producing K. pneumoniae* panres and *A. baumannii*	Surgical site
132–146	Meropenem Tigecycline	2 gr/6 hour 100 mg/12 hour	Rectal swab *KPC-producing K. pneumoniae* panres Blood cultures negative	Intestine Blood stream

*LD* loading dose, *KPC Klebsiella pneumoniae* carbapenemase*,* *MDR* multidrug-resistant, *MU* million, *panres* panresistant

He had a short-lasting improvement, with fever, abdominal pain, and vomiting relapsing after a few days; a repeated CT scan of his abdomen revealed suprapubic and left ischiatic abscesses at surgical sites, as well as dilated bowel due to paralytic ileus. Surgical debridement was performed, followed by vacuum-assisted closure (VAC) therapy [16]. Intraoperative microbiological sampling revealed multiple CRKP isolates, with a worsened resistance profile, including colimycin (colistin) resistance. He improved and a control radiographic (RX) scan of his thorax was negative; bronchoalveolar lavage (BAL) sampling, however, confirmed persistence at low bacterial load of CRKP. After 21 days, colimycin was withdrawn. However, 4 days later, he had fever and severe leukocytosis; he relapsed with acute renal failure: creatinine 2.53 mg/dL and acute kidney injury (AKI) stage 2 according to the Kidney Disease: Improving Global Outcomes classification (KDIGO) [17]. His PCT levels rose above 100 ng/ml, his lactate was 3.3 mmol/L, and his SOFA score was 10. After further blood sampling for blood cultures and multiplex polymerase chain reaction (PCR; Magicplex™ Sepsis Test, Seegene), colimycin and anidulafungin were restarted with the addition of rifampicin (Table 1). Multiplex PCR revealed *A. baumannii* and *Candida albicans*. To support septic shock recovery, two extracorporeal hemoperfusion devices were used: the Polymyxin B-Immobilized Cartridge (Toraymyxin® PMX 20-R, Toray Medical, Tokyo, Japan), allowing endotoxin removal and coupled plasma filtration adsorption (CPFA; CPFA® LYNDA®, Bellco, Mirandola, Italy), a hydrophobic resin with high affinity for many inflammatory mediators. Within 3 days, two Polymyxin B and three CPFA treatments were overall delivered. Clinical improvement ensued; his PCT fell to 19.25 ng/ml and serum creatinine to 1.82 mg/dL. Microbiological samples yielded: MDR *A. baumannii* and CRKP from tracheal aspirate, *C. albicans* and MDR *A. baumannii* from blood cultures, and *A. baumannii* and panresistant CRKP from wound swabs. In spite of septic shock reversal, he remained febrile with diarrhea and worsening anemia in the next 2 weeks. Control blood cultures (three lots) were persistently positive for *C. Albicans, A. baumannii* and CRKP. Ophthalmoscopy revealed retinal involvement, so that sequential therapy with liposomal amphotericin B was started. In the following weeks frequent vomiting ensued, impeding any enteral nutrition; his abdominal pain increased, paralleled by a palpable mass in his right flank. Colonoscopy revealed patchy serpiginous ulcers; a repeated CT of his abdomen revealed a periappendicular mass. Based on such data, after repeated multidisciplinary consults, an exploratory laparotomy was at last performed and an inflammatory pseudotumor of his right colon was diagnosed and resected with ileostomy. Soon after surgery, his blood cultures turned negative. Colimycin was interrupted. Once more, septic shock relapsed 4 days later. Leukocytosis, hypotension, PCT >100 ng/mL, renal failure, and lactate 4.0 mmol/L led to a SOFA score of 11; creatinine zenith was 4.73 mg/dL. Combination antibiotic therapy was modified (see Table 1) and a single Polymyxin B extracorporeal hemoperfusion cycle was repeated. Blood cultures were persistently positive for CRKP. After interdisciplinary consultation, removal of pelvic synthesis media was anticipated. Shortly after surgery, he improved; 3 weeks later, microbiological and biochemical evidence of infection resolved, as well as gastric atonia. Combination therapy was continued for 56 days (Table 1). He was transferred to the orthopedic ward after 146 days of ICU stay and later to rehabilitation. At present, he is doing well at home able to walk.

## Discussion

Our patient had a severe abdominal and pelvic trauma which received immediate intensive transfusional and surgical support [12, 14]. His long-lasting watery diarrhea was probably trauma related: his first CT scan revealed signs of post-traumatic right colon injury, which probably caused altered intestinal motility, ulcerative colitis, and ultimately cecal inflammatory pseudotumor [18–20]. Due to trauma, he developed two bloodstream seeding sites – the intestine and pelvic surgical sites – which we could not eliminate until late in his clinical course; this influenced the heavy and inevitable selection of MDR bacteria [4, 21]. So we needed to treat his relapsing septic episodes to enable his survival throughout the time needed for appropriate and definitive infection source control. He did in fact survive a series of five septic shock episodes without residual signs of kidney or other organ failure at discharge. We used a complex, costly, and articulated strategy to achieve this aim, which we find totally justified in this case and worth adequate consideration [22, 23].

**Fig. 2:**
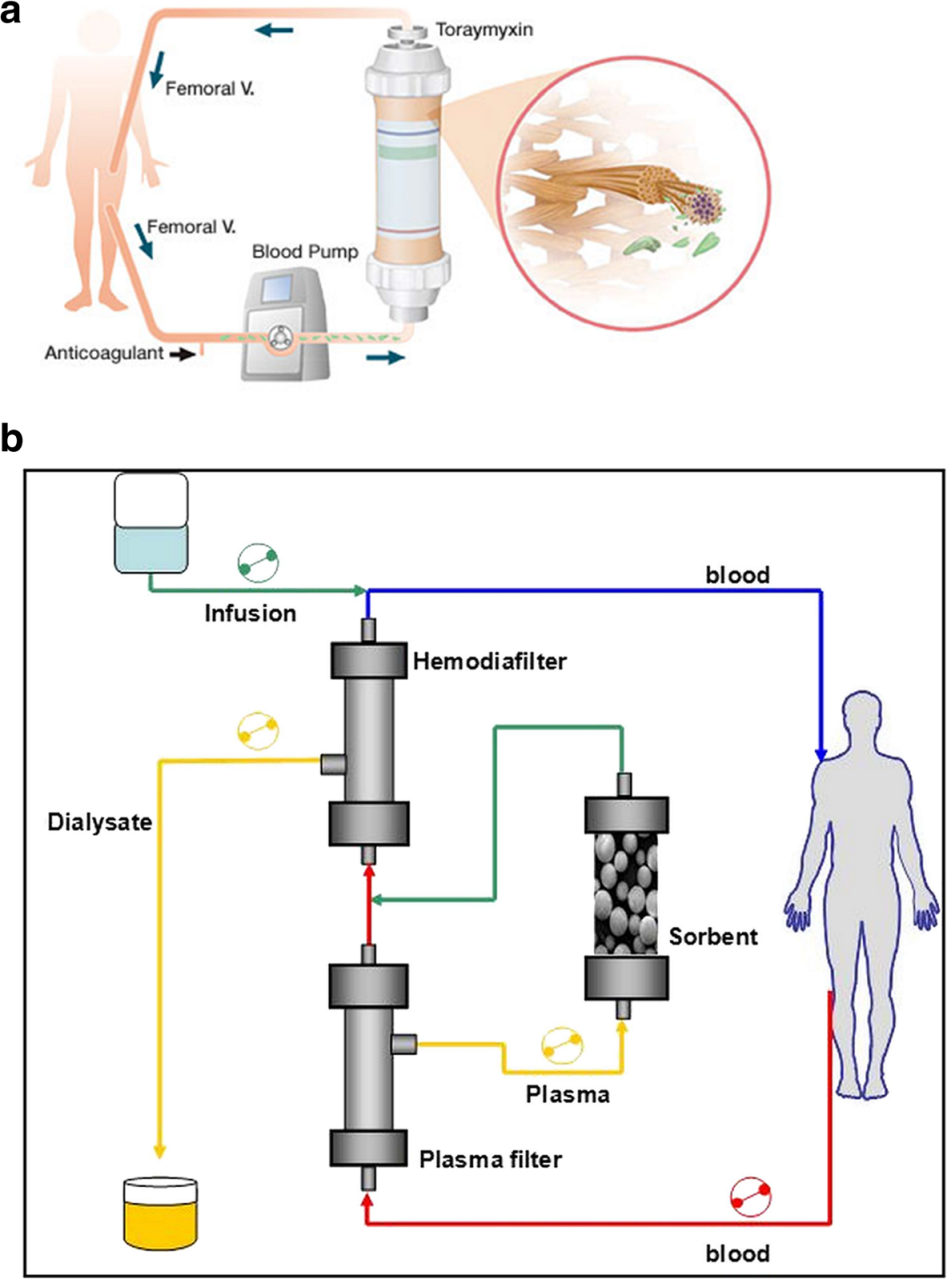
**a** Toraymyxin hemoperfusion scheme (duration 2 hours), as accessed at www.estor.it. **b** Coupled plasma filtration adsorption schematic diagram, as accessed at www.bellco.net. *V.* vein

In this strategy, early deployment of blood purification techniques to not only support renal function during the acute phases of renal overload, but also to remove endotoxins and cytokines, had a key role. Alongside extracorporeal therapies for the treatment of renal failure, new extracorporeal depurative techniques have been developed for the removal of endotoxin and inflammatory mediators [24]. Toraymyxin® is the reference for the treatment of patients with endotoxic septic shock unresponsive to conventional therapies, with a high endotoxin removal capacity per hemoperfusion treatment. Toraymyxin® was designed to adsorb endotoxin, but it probably adds other mechanisms of immunomodulation as direct adsorption of some inflammatory mediators (Fig. 2a) [25]. Another technique, named CPFA, has been proposed to non-specifically remove both proinflammatory and anti-inflammatory mediators [26]. This technique consists of a combination of filters and a resin cartridge to remove a number of different cytokines including tumor necrosis factor-α, interleukin (IL)-6 and IL-10, while simultaneously providing continuous renal replacement therapy (CRRT) for renal/fluid support (Fig. 2b). Application of CPFA has been demonstrated to reduce hospital mortality in patients with septic shock in ICUs [27]. The Compact 2 study, an ongoing multicentric trial, may shed further light on this point [24]. In the case of our patient, with relapsing episodes of septic shock in the absence of timely infectious source control, we decided to sequentially use both extracorporeal depurative techniques in the search of optimal control of both mechanisms of shock pathogenesis: that is, endotoxin release from persisting infectious foci, and cytokine production due to host response.

It is important to underline the relevant role that interdisciplinary discussion had in this patient. In particular, microbiological evidence of persistent bacterial and fungal translocation suggested a laparoscopic evaluation, which surgeons had first denied on the basis of CT and clinical evidence. Similarly, the interplay with orthopedic surgeons led to the choice of the first reasonable slot for synthesis media removal. Efforts to use the best of antimicrobial combination therapy for sepsis control [7, 28] were the fruit of interplay with infectologists. CRKP has become a major hospital pathogen worldwide, and infections due to this organism have been associated with high mortality, especially in cases like ours that harbor panresistant strains [29]. In our case, it is of note that, in the wait for new antimicrobial options, the combination of two carbapenems proved partially effective on panresistant strains [28, 29].

The room and theatre of all these advanced procedures was the ICU. This was relevant for our patient, and should prompt internists to evaluate once more the role of intensive care in the management of patients with sepsis.

## Conclusions

High quality intensive assistance for complex cases involving patients with sepsis may ensue through the active interplay of ICU physicians and multiple specialists to define a rational sequence of interventions aimed to prevent multiple organ failure until control of the infection source is complete.

## Abbreviations

AKI: Acute kidney injury
APACHE: Acute Physiology and Chronic Health Evaluation
ATLS: Advanced Trauma Life Support
BAL: Bronchoalveolar lavage
CPFA: Coupled plasma filtration adsorption
CRKP: Carbapenem-resistant *Klebsiella pneumoniae*
CRP: C-reactive protein
CRRT: Continuous renal replacement therapy
CT: Computed tomography
ICU: Intensive care unit
IL: Interleukin
ISS: Injury Severity Score
KDIGO: Kidney Disease: Improving Global Outcomes
KPC: *Klebsiella pneumoniae* carbapenemase
MDR: Multidrug-resistant
PCR: Polymerase chain reaction
PCT: Procalcitonin
PIRO: Predisposition, Infection, Response, and Organ Dysfunction
RX: Radiographic
ScVO_2_: Central venous saturation of oxygen
SOFA: Sequential Organ Failure Assessment
VAC: Vacuum-assisted closure
